# Those who speak survive: the value of the verbal component of GCS in trauma

**DOI:** 10.1007/s00068-022-02153-0

**Published:** 2022-11-06

**Authors:** Arif Alper Cevik, David Olukolade Alao, Eman Alyafei, Fikri Abu-Zidan

**Affiliations:** 1 Department of Internal Medicine, Emergency Medicine Section, College of Medicine and Health Sciences, United Arab Emirates University, 17666 Al Ain, United Arab Emirates; 2 Emergency Department, Tawam Hospital, Al Ain, United Arab Emirates; 3 Emergency Department, Al Ain Hospital, Al Ain, United Arab Emirates; 4 Department of Surgery, College of Medicine and Health Sciences, United Arab Emirates University, Al Ain, United Arab Emirates

**Keywords:** Major trauma, GCS components, Survival prediction

## Abstract

**Aim:**

To evaluate the value of the individual components of GCS in predicting the survival of trauma patients in the Emergency Department.

**Methods:**

Trauma patients who were admitted for more than 24 h or died after arrival at Al-Ain Hospital from January 2014 to December 2017 were studied. Children < 16 years, elderly > 80 years, patients with facial injuries, those intubated in the ER, and those with missing primary outcomes were excluded. Demography, vital signs, Glasgow Coma Scale (GCS), GCS components, Injury Severity Score (ISS), head AIS, and death were compared between those who died and those who survived. Factors with a *p* value of < 0.1 were entered into a backward likelihood logistic regression model to define factors that predict death.

**Results:**

A total of 2548 patients were studied, out of whom 11 (0.4%) died. The *verbal component of* GCS (*p* < 0.001) and the ISS (*p* = 0.047) were the only significant predictors for death in the logistic regression model. The AUC (95% CI) of the GCS-VR was 0.763 (0.58–0.95), *p* = 0.003. The best point of GCS-VR that predicted survival was 5, having a sensitivity of 97%, a specificity of 54.5%, positive predictive value of 99. 8%, negative predictive value of 7.3%, and likelihood ratio of 2.13.

**Conclusion:**

In general trauma patients, acute trauma care professionals can use GCS-VR to predict survival when clinical condition permits instead of the total GCS score or ISS.

## Introduction

The Glasgow Coma Scale (GCS) is widely used to assess a trauma patient's level of consciousness. It consists of three components: eye, motor, and verbal responses; each is scored on a specific scale. The sum is used to assess the overall responsiveness of the patient [1]. It is used to triage patients in the prehospital setting and to predict patients' outcomes [2, 3]. Despite its usefulness, it has multiple limitations. Calculating the total GCS score requires time, especially in the prehospital setting, where quick decisions on transferal to trauma centers are needed [4]. Furthermore, although the score is relatively reliable when used by experienced medical personnel [3], poor physician knowledge and interrater reliability have been reported [5–8]. In addition, different combinations with the same total score have been shown to have different mortality outcomes [9].

Previous studies investigated whether a single component of GCS can predict survival as adequately as the total GCS. Some reported clinical equivalence in the prediction ability of the total GCS compared with the motor component [3, 4, 10–12]. The motor component is the most challenging for assessors [13]. Most studies on the role of single components of GCS as a predictor of outcomes are on traumatic brain injury. The reduced GCS in trauma patients is often attributed to traumatic brain injury, but it can be caused by other conditions such as hypoxia, hypovolemia, alcohol intoxication or drug abuse. We aimed to evaluate the value of the individual components of GCS in predicting the survival of trauma patients in the emergency department (ED).

## Methods

This is a post hoc analysis of previously published data on trauma patients in Al Ain City [14]. We abstracted data of all trauma patients from Al Ain Hospital that were included in the Abu Dhabi trauma registry from January 2014 to December 2017. The trauma registry prospectively collects data on all trauma patients admitted for more than 24 h or who died at the Emergency Department or hospital. Seven hospitals, including Al Ain Hospital, the major trauma center for the Al Ain region of Abu Dhabi, contribute data to the registry. Data are entered into the registry database by trained research nurses and are validated centrally by the department of health of Abu Dhabi for quality assurance.

### Ethical approval

Al-Ain Hospital Human Research Ethics Committee, Al Ain, United Arab Emirates, approved the study (approval number AAHEC-03-20-008). Written informed consent to use patients' data was obtained from patients or next of kin. All patients' identifiers were fully anonymous during the analysis.

### Study setting and population

Al-Ain Hospital is the major trauma center for the Al Ain region of Abu Dhabi. It has a population of 766,000 inhabitants. We included all trauma patients who were admitted for more than 24 h or who died in the Emergency Department or the hospital from January 2014 to December 2017. We have excluded patients who may have difficulty talking as a baseline. Exclusion included patients with an age of more than 80 years (*n* = 52) or less than 16 years (*n* = 491), facial injuries (*n* = 403), and those intubated in the ED (*n* = 15). Ten patients did not have the final clinical outcome and were excluded. The study population is a subset of our previously published trauma registry data [14].

### Study design

This is a retrospective analysis of prospectively collected data of Al Ain Hospital Trauma Registry.

### Studied variables

Patients' Demographics (age, sex, nationality), vital signs (systolic blood pressure, heart rate, respiratory rate, oxygen saturation), abbreviated injury severity (AIS) of the head injury, total GCS, GCS components (eye-opening—GCS-EO, verbal response—GCS-VR, motor response—GCS-MR), Injury Severity Score (ISS), and in-hospital mortality were analyzed.

### Statistical analysis

We divided patients into two groups: those who survived and those who died. The Mann–Whitney *U* test was used to compare continuous or ordinal data between the two independent groups. Fisher's exact test was used for the comparison of categorical data of the two independent groups. Continuous and ordinal data were presented as median 25th–75th inter-quartile range (IQR), while categorical data were presented as numbers (%). Factors in the univariate analysis with a *p* value of less than 0.1 were entered into a direct logistic regression model to define factors that predict survival. The receiver operating characteristic (ROC) curve was applied to the logistic regression model. Statistical Package for the Social Sciences (IBM-SPSS version 26, Chicago, IL) was used for analyses, and *a p value* of less than 0.05 was accepted to be significant.

## Results

There were 3519 trauma patients in the registry during the study period. Most injuries were caused by falls (*n* = 936, 26.6%) and road traffic collisions (*n* = 921, 26.2%). Injuries occurred mainly at home (*n* = 1346, 39%) and on the street/highway (*n* = 989, 28.7%). A total of 2548 patients were included in this study and analyzed. The patients' median (IQR) age was 34 years (26–44), and 83.7% were males. The median (IQR) GCS and ISS were 15 (15–15) and 4 (4–9), respectively. Three hundred and eighteen patients (12.5%) had head injuries, 107 (4.2%) had neck injuries, 390 (15.3%) had chest injuries, 207 (8.1%) had abdominal and pelvic injuries, 426 (16.7%) had spine injuries, 962 (37.8%) had upper limb injuries, and 959 (37.6%) had lower limb injuries. None had facial injuries because those were excluded from the study. Eleven patients (0.4%) died.

**Fig. 1:**
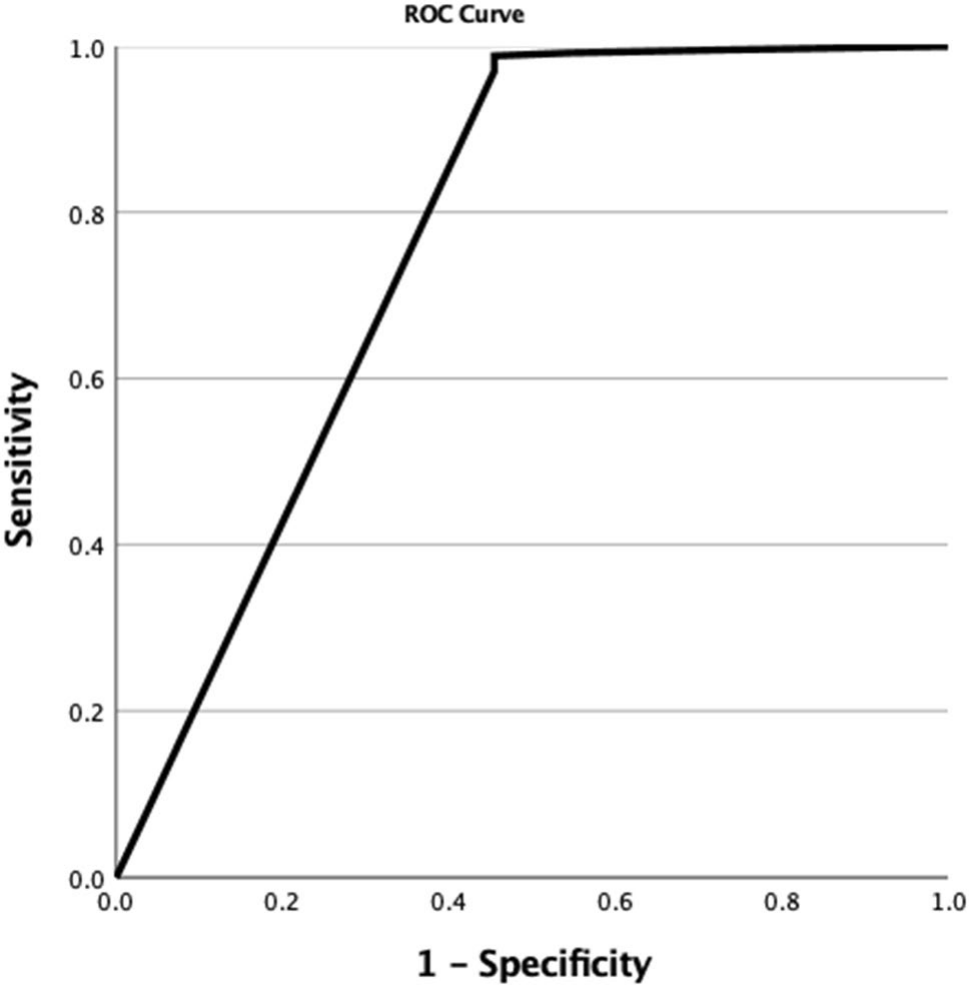
Receiver operating characteristic (ROC) curve of the GCS verbal response (GCS-VR) that predicted survival in the logistic regression model in 2527 trauma patients. Area under the curve values for GCS-VR was 0.76

**Fig. 2:**
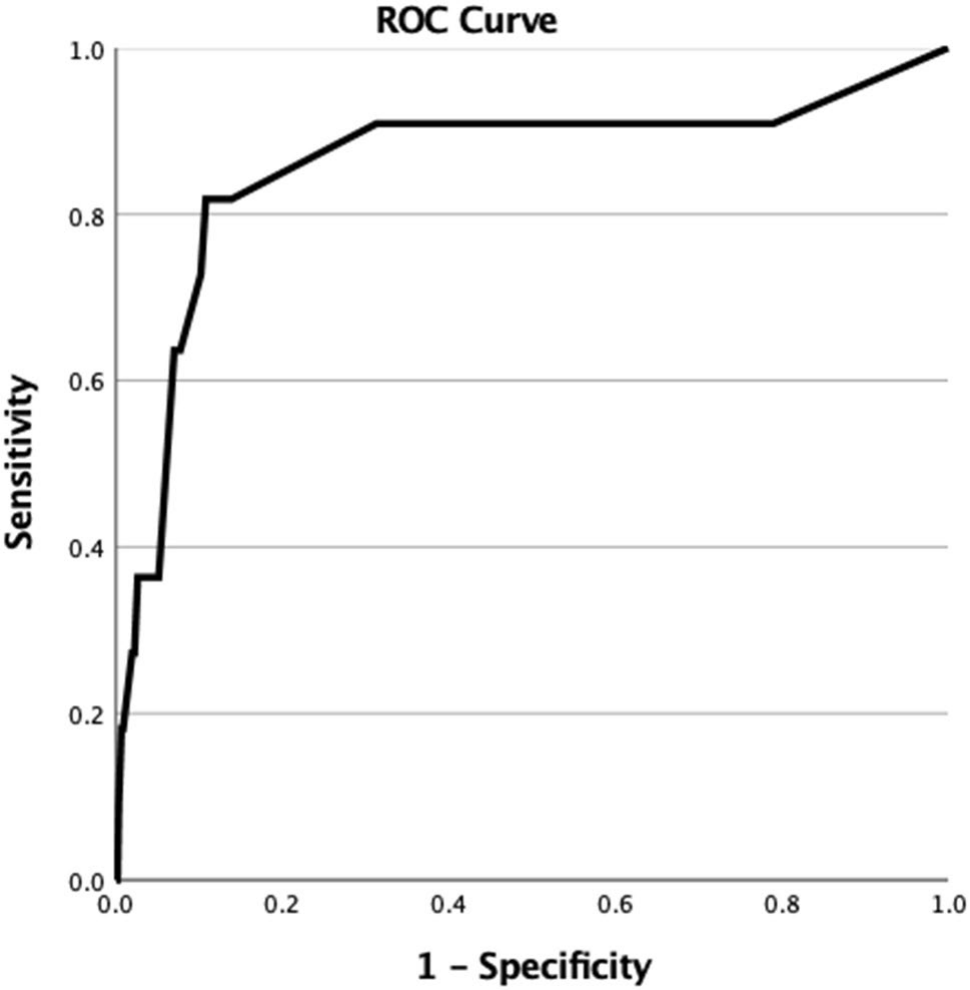
Receiver operating characteristic (ROC) curve for Injury Severity Score (ISS, blue line) that predicted mortality in the logistic regression model in 2504 trauma patients. The area under the curve was 0.86

The patients who survived had significantly lower median (IQR) heart rate, 85 (77–94) vs 97 (80–100) beats/min, *p* = 0.04; and lower ISS, 4 (4–9) vs 16 (12–25), *p* < 0.001, compared with those who died. Patients who survived had significantly higher median (IQR) GCS: 15 (15–15) vs 10 (5–15), *p* < 0.001. Table 1 shows the univariate analysis of patient demographics and physiological parameters by outcome. Variables that had a *p* value of less than 0.1 were entered into the logistic regression model. Table 2 shows the logistic regression analysis results. The model was highly significant (*p* < 0.001, Nagelkerke *R*^2^ = 0.282). GCS-VR (*p* < 0.001) and ISS (*p* = 0.047) were the only significant predictors in the model. Figures 1 and 2 show GCS-VR and ISS ROC curves, respectively.

**Table 1:** Patient demographics and physiological parameters by outcome

Variable	Patients who survived *N* = 2537	Patient who died *N* = 11	*p* Value
Age	34 (26–44)	39 (27–57)	0.19
Sex			0.26
Male	2125 (83.8%)	8 (72.7%)	
Female	412 (16.2%)	3 (27.3%)	
Nationality			0.51
UAE	372 (14.7%)	1 (9.1%)	
Non-UAE	2165 (85.3%)	10 (90.9%)	
Vitals			
Systolic blood pressure (mmHg)	134 (123–147)	120 (112–146)	0.31
Heart rate (per minute)	85 (77–94)	97 (80–100)	0.04
Respiratory rate (per minute)	18 (18–19)	22 (16–28)	0.07
Sat O_2_%	99 (98–100)	99 (96–100)	0.8
Head injury	312 (12.3%)	6 (54.5%)	< 0.001
GCS 13–15	2485 (12.3%)	6 (54.5%)	< 0.001
GCS 9–12	312 (12.3%)	6 (54.5%)	< 0.001
GCS 3–8	312 (12.3%)	6 (54.5%)	< 0.001
GCS total	15 (15–15)	10 (5–15)	< 0.001
GCS level			< 0.001
13–15	2485 (98.3%)	5 (27.3%)	
9–12	24 (0.9%)	3 (27.3%)	
3–8	18 (0.7%)	3 (45.5%)	
GCS—eye	4 (4–4)	3 (1–4)	< 0.001
GCS—verbal	5 (5–5)	3 (1–5)	< 0.001
GCS—motor	6 (6–6)	5 (3–6)	< 0.001
AIS head	0 (0–0)	3 (0–4)	< 0.001
ISS	4 (4–9)	16 (12–25)	< 0.001

Results in table were given as median (IQR, 25–75 percentile range) and number (percentage) where appropriate. *p* Value calculated by using nonparametric tests (Mann–Whitney *U* and Fisher exact test) where appropriate

*AIS* Abbreviated Injury Scale; *GCS* Glasgow Coma Scale; *ISS* Injury Severity Score, *UAE* United Arab Emirates

**Table 2:** Logistic regression model defining significant predictors for death of patients with trauma (*n* = 2548)

Variable	Estimate	SE	Wald test	*p* value	OR		95% CI
GCS-VR	− 1.012	0.241	17.626	< 0.001		0.363	0.227–0.583
ISS	0.072	0.036	3.953	0.047		1.075	1.001–1.155
Constant	− 1.598	1.319	1.469	0.226		0.202	

*SE* standard error; *OR* odds ratio; *CI* confidence interval; *GCS-VR* GCS verbal response; *ISS* injury severity score

Areas under the curves (AUC) of total GCS predicting survival and ISS predicting death were 0.76 and 0.86, respectively. AUCs for GCS-EO, GCS-MR and GCS-VR were 0.77, 0.77 and 0.76, respectively. The AUC (95% CI) of the GCS-VR was 0.76 (0.58–0.95) which was significantly higher compared with the null hypothesis 0.5 AUC (*p* = 0.003, asymptotic nonparametric comparison). The best point of GCS-VR that predicted survival was 5, with a sensitivity of 97%, a specificity of 54.5%, positive predictive value of 99.8%, negative predictive value of 7.3%, and a likelihood ratio of 2.13.

## Discussion

This study found that GCS-VR and ISS were the only independent factors predicting survival in trauma patients in the logistic regression model, with GCS-VR having a higher significance. The eye and motor components of GCS were not significant in predicting trauma survival in the model.

The ISS is a valuable predictor of trauma outcomes [15, 16]. Despite the wide use of ISS in trauma patients, various drawbacks exist. First, the variables required to calculate ISS are unavailable in the prehospital and the ED. Second, the ISS can underestimate injury severity and mortality of patients with multiple organ injuries since it considers only one injury per body region up to a maximum of three regions [17]. Also, the ISS is an anatomical scoring which neglects the physiological differences and different interactions that patients express in response to trauma [18]. In addition, low inter-observer agreement of ISS has been reported [19]. Finally, ISS requires training and time to calculate [20]. These factors limit the utility of ISS as a survival predictor in the early phase of trauma care. The GCS is better in this regard.

The GCS was first described in head trauma patients in 1974 [21]. It became widely used as an objective method for predicting the severity and mortality of various clinical conditions, including general trauma [16, 22, 23]. While it is considered simpler than ISS, GCS still requires the assessment of complex eye, motor, and verbal components. These may lead to calculation errors and low interrater agreement [24, 25]. In addition, the assessment of the GCS components may be highly subjective and not easily reproducible, adding to interrater variability and poor reliability. Another downside of the GCS assessment is that not all components contribute to the overall score discrimination [24]. In patients with GCS below 8, further reduction in the sum score reflects changes in the motor component as the eye and verbal components would have achieved a flooring effect [9]. Furthermore, in patients with scores between 9 and 15, the eye and verbal responses are the main determinants of changes as the motor component reaches a ceiling effect [9, 21, 24]. Similarly, motor responses can be affected by local extremity injuries and spinal cord or peripheral nervous system injuries. The eye component of the GCS may be misleading in some clinical scenarios. For example, spontaneous open eyes do not necessarily mean the patient is fully awake. Furthermore, the verbal response is difficult to assess in patients with endotracheal intubation, maxillofacial injuries, children, and the elderly with dementia. We excluded these groups of patients from our study, limiting our analysis to those for whom GCS can be reliably assessed.

Researchers have looked for alternatives to replace the total GCS score to circumvent these challenges. Single component studies on motor GCS, Simplified Motor Score, and ‘patient doesn't follow command’ have reported no difference in severity and mortality predictions in trauma patients compared with the total GCS. Therefore, they have recommended the motor component as a substitute for the total GCS [3, 4, 25]. Despite the advantages of replacing GCS with only the motor component, it has several limitations.

Gill et al. reported a significant interrater variability in the motor components of the GCS among emergency physicians [6]. The variability is mainly attributed to the difficulty of differentiating 'localized response' and 'abnormal flexion response' [26]. In addition, differentiation between 'abnormal flexion' and 'normal flexion' may also be difficult [27]. Furthermore, a study done on ED professionals found that the verbal component has the highest accuracy compared with the other two components of the GCS [28]. The verbal, unlike the motor, is the first component to be affected in patients with mild to moderate TBI [9]. The median (IQR) of the GCS in our study population was 15 (15–15), while the median (IQR) for ISS was 4 (4–9), indicating that majority of our patients had mild to moderate injury severity. This may explain the findings in our prediction model. This makes the verbal component a very useful triage tool in the early phase of trauma care.

## Limitations of this study

We recognize that our study has some limitations, and our results should be interpreted in light of this. *First*, the registry consists of trauma patients admitted to a single center. Although Al Ain Hospital is the only trauma center in the city and manages around 80% of trauma patients, the registry does not capture the data for patients who died at the scene. Hence, our study may have underreported the mortality rate. *Second*, the median ISS was 4, and the median GCS was 15, indicating that most of the patients had minor to moderately severe injuries, which may also explain the low mortality rate. Our findings may not be generalized to those in whom the GCS cannot be assessed, such as intubated patients, those with maxillofacial injuries, elderly, and children. *Third*, the number of events (death) was very small. Only eleven patients (0.4%) died, which explains the wide range of confidence interval of the AUC of the GCS-VR, which decreases the confidence of finding the real AUC of our study. *Forth*, although the logistic regression was highly significant, its *R*^2^ was only 0.28, indicating that the GCS-VR and ISS explain only 28% of the variation of the model. *Finally*, we did not have data on alcohol intoxication in our study. The incidence of alcohol intoxication among trauma patients in our city is very low due to cultural and religious norms; only 2% of admitted car occupants trauma patients in our hospital were documented to be under the influence of alcohol [29]. The impact of alcohol on our findings is, therefore, minimal.

## Conclusions

Our study showed that emergency department admission GCS-VR was the most significant factor that predicts survival in trauma patients. Acute trauma care professionals can use the initial GCS-VR to predict the survival of general trauma patients in the ED when clinical condition permits. The patient who 'speaks' on arrival in ED has a high probability of survival.

## Data Availability

No additional data are available for the reader. Further data can be provided to the journal's Editor upon request.
